# Laparoscopic Versus Open Caudate Lobe Resection: A Systematic Review with a Meta-Analysis of Comparative Studies

**DOI:** 10.3390/jcm14134421

**Published:** 2025-06-21

**Authors:** Gianluca Cassese, Fabio Giannone, Celeste Del Basso, Mariantonietta Alagia, Marco Lodin, Igor Monsellato, Marco Palucci, Federico Sangiuolo, Gabriela Del Angel Millan, Fabrizio Panaro

**Affiliations:** 1 Division of Hepato-Pancreato-Biliary, Oncologic and Robotic Surgery, Azienda Ospedaliero-Universitaria SS. Antonio e Biagio e Cesare Arrigo, 15121 Alessandria, Italy; gianluca.cassese@uniupo.it (G.C.); celeste.delbasso@ospedale.al.it (C.D.B.); mariantonietta.alagi@ospedale.al.it (M.A.); marco.lodin@ospedale.al.it (M.L.); igor.monsellato@ospedeale.al.it (I.M.); marco.palucci@ospedale.al.it (M.P.); federico.sangiuolo@ospedale.al.it (F.S.); delangelmillang@gmail.com (G.D.A.M.); fabrizio.panaro@uniupo.it (F.P.); 2 Department of Research and Innovation (DAIRI), Azienda Ospedaliero-Universitaria SS. Antonio e Biagio e Cesare Arrigo, 15121 Alessandria, Italy; 3 Department of Health Sciences, School of Medicine, University of Eastern Piedmont “Amedeo Avogadro”, 28100 Novara, Italy

**Keywords:** laparoscopic liver resection, caudate lobe, segment one

## Abstract

**Background:** Liver resection of a caudate lobe is a challenging procedure in both open and minimally invasive approaches. The reason is mainly related to its anatomical position: segment 1 (S1) lies on the inferior vein cava, behind the main and the left portal veins, and below the hepatic veins. This meta-analysis aimed to assess the results of laparoscopic liver resection (LLR) versus open liver resection (OLR) for S1 resection. **Methods:** Available literature up to June 2024 was retrieved from the Medline and Embase databases. A systematic review with a meta-analysis was carried out to investigate the safety and efficacy of LLR for the S1 segment. **Results:** Six studies including 292 patients (LLR: *n* = 132; OLR: *n* = 160) were selected for the meta-analysis. The OLR cohort showed higher estimated blood loss (EBL) (MD: 140.1, 95% CI 49.3–130.8; *p* = 0.011) and longer length of hospital stay (MD: 3, 95% CI 1.8–4.2; *p* = 0.001). No differences in severe postoperative morbidity, overall morbidity, R1 resection rates, transfusion rates, operative time, and duration of Pringle maneuvers were shown. **Conclusion:** LLR for lesions located in S1 is safe and effective and may be associated with lower EBL and shorter length of stay than OLR. Further larger prospective studies are needed to confirm such results.

## 1. Introduction

Liver surgery is a cornerstone treatment for most early-stage liver malignancies, as well as for some symptomatic benign lesions. Some types of liver resection are considered technically challenging in both open and minimally invasive approaches. In particular, liver resection of segment 1 (S1) is a technically demanding procedure in both open and laparoscopic approaches [1]. The difficulty is related to the complex anatomy of S1 due to the close relationships with the inferior vena cava (IVC), the main and the left portal veins, as well as to the middle and right hepatic veins that constitute the upper limit of its intrahepatic part [2]. Nonetheless, S1 has the peculiarity of being independent with its own vascular and biliary anatomy and of having many possible anatomical variations.

Laparoscopic liver resection (LLR) of segment 1 (S1), also called isolated laparoscopic caudate lobectomy, is considered even more challenging than the open procedure [3]. Since the first LLR research was published in 1991 by Reich et al., its spread has been slower than other types of surgeries [4]. The main concerns were the technical difficulty in hilar dissection, the complexity of parenchymal transection, the risk for bleeding, the difficulty in achieving adequate resection margins, and the complexity of treating patients with underlying hepatopathy [5]. During the last decades, growing evidence about safety and effectiveness of LLR has been published, showing how LLR may be a viable option for both benign and malignant liver lesions [6,7,8,9]. Subsequently, LLR has been shown to be able to reduce the length of hospital stay, the level of blood loss, the rates of transfusions, and the postoperative pain of lesions located in different liver segments [10,11,12,13]. With the implementation of laparoscopic experience and expertise, experienced hepatobiliary surgeons in referral HPB centers are currently performing more and more complex LLR [14].

Subsequently, during the last years, LLR of S1 is being increasingly performed and reported in literature thanks to a wider adoption of minimally invasive liver surgery (MILS), as well as to the encouraging results on MILS in lesions with difficult locations [12]. Indeed, LLR of S1 is still considered one of the most challenging laparoscopic surgeries, being part of the “difficult” posterosuperior segments, together with segments IVa, VII and VIII [10,15]. Thus, LLR for S1 is currently performed only in experienced centers, with few studies available in the literature reporting its outcomes.

Since no systematic reviews with meta-analyses of comparative studies are available to date, this study was aimed at assessing the postoperative results of LLR vs. OLR of caudate lobes.

## 2. Materials and Methods

### 2.1. Literature Search Strategy

The meta-analysis was conducted according to the Meta-analyses Of Observational Studies in Epidemiology (MOOSE) and the Preferred Reporting Items for Systematic Reviews and Meta-Analyses (PRISMA) guidelines [16,17] after being registered in PROSPERO. The search was performed over the Embase and Medline electronic databases to find studies published up to September 2024. An additional search was carried out to find potentially relevant studies from the reference list of the included studies. The search strategy involved using Boolean connectors, including the following terms: (posterior OR postero* OR difficult OR caudate OR spiegel) AND (hepato OR hepat* OR liver) AND (resection OR hepatectomy) AND ((minimally AND invasive) OR laparoscop* OR laparoscopic).

Two reviewers (FG and GC) independently screened the titles and abstracts retrieved from the research. Then, the full texts were retrieved for final analysis and data extraction.

### 2.2. Study Selection Criteria

Liver resections were defined following the 2000 Brisbane nomenclature [18]. Two reviewers independently selected the articles to include in the meta-analysis according to the following inclusion criteria: (1) comparative studies investigating OLR versus LLR for tumors located in S1 and (2) available data about intra- and post- operative results. The exclusion criteria were as follows: (1) articles not written in the English language and (2) articles including fewer than 10 patients.

### 2.3. Data Extraction

The primary outcomes were the postoperative results in terms of postoperative hospital stay, postoperative morbidity, major morbidity, reoperation rate, 30-day mortality, 90-day mortality, R0 resection rate, and 30-day readmission rate.

Data regarding the aforementioned results, as well as the first author, country, article type, year of publication, number of patients, age, sex, baseline patient characteristics, intraoperative outcomes (operative time, intraoperative blood loss, blood transfusion rate, Pringle maneuver application rate, and Pringle maneuver application time), were retrieved from two reviewers (FG and GC). A third researcher (FP) was questioned in case of inconsistencies. In case of propensity score-matched studies, data were extracted from the matched groups to reduce selection bias.

### 2.4. Quality Assessment and Publication Bias

The quality of each study was evaluated by using the risk of bias in non-randomized studies of interventions (ROBINS-I) tool. Subsequently, the *robvis* web app was used to display the results of the quality assessment [19]. By using this tool, each one of the included studies was appraised by exploring seven domains, and each domain was judged to possibly have a low, moderate, serious, or critical risk of bias or no information. If at least one domain was assessed to be at serious or critical risk of bias, the corresponding study was defined as a study with a serious/critical risk of bias. The quality of the included studies was evaluated independently by two reviewers (FG and GC), involving the third reviewer (FP) in case of inconsistencies.

Publication bias was evaluated by analyzing the symmetry of funnel plots.

### 2.5. Statistical Analysis

A fixed-effect model was used to pool data in case of low or absent existing heterogeneity, using a Mantel–Haenszel method to calculate the weights of the included studies with binary outcomes [20]. A random-effect model was used in case of higher heterogeneity, with the Paule–Mandel estimator method being used to calculate the variance distribution given the binary and continuous effect size data [21]. Effects sizes were evaluated through mean differences (MD) for continuous variables or odds ratio (OR) for dichotomous ones together with their relative 95% confidence interval (CI).

The presence of statistical heterogeneity was analyzed using the Cochrane Q test and quantified through the inconsistency index (I^2^), with values of 25, 50 and 75% for I^2^ considered as thresholds for low, moderate and substantial heterogeneity, respectively. A sensitivity analysis was performed by checking for outliers and influential cases when I^2^ was greater than 50%.

Assessment of publication bias was performed by using funnel plots. The risks of bias of the included studies were analyzed through the “methodological quality the risk of bias in non-randomized studies—of interventions” (ROBINS-I) tool, by using the *robvis* web tool [22]. A *p*-value < 0.050 was considered as statistically significant.

R software version 4.4.1 (2019, The R Foundation for Statistical Computing) was used for statistical analyses.

## 3. Results

### 3.1. Study Selection

**Figure 1:**
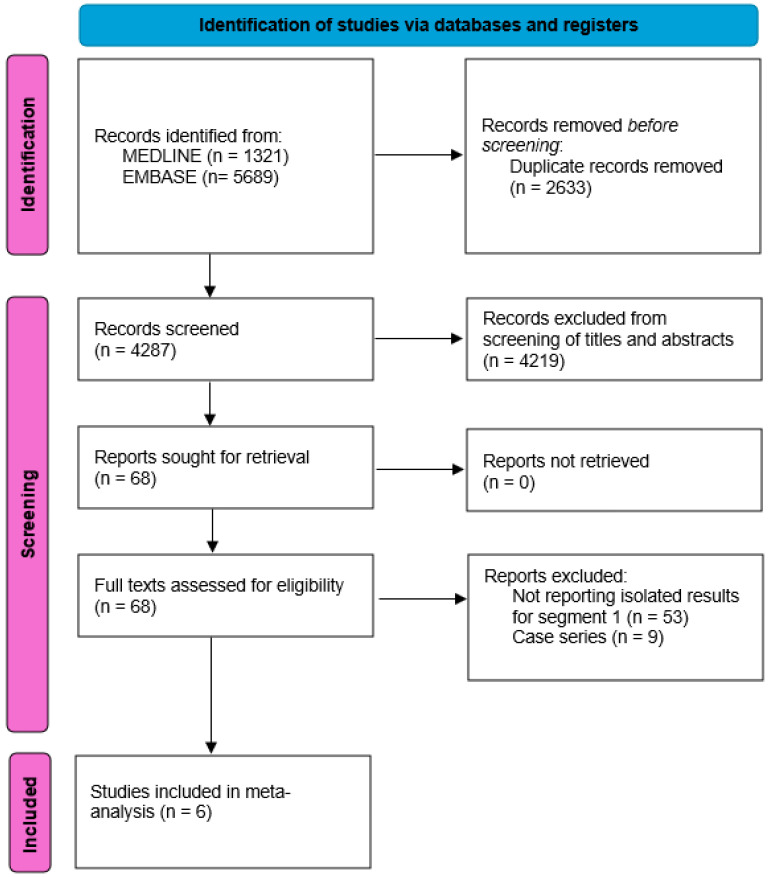
PRISMA flow diagram illustrating the study selection.

The PRISMA flowchart for the screening of the included articles can be found in Figure 1. A total of six studies met the inclusion criteria and were selected for the meta-analysis, including a total of 292 patients undergoing liver resection of S1.

### 3.2. Study and Patient Characteristics

The meta-analysis included a total of six comparative studies, with 292 patients (OLR: *n* = 160; LLR: *n* = 132) [23,24,25,26,27,28]. The studies were published between 2020 and 2022, with inclusion periods ranging from 2004 to 2020. All studies were retrospective, with four out of six being based on a propensity score matching (PSM) comparison.

Indications for surgery included malignant diseases in 61.8% of the OLR (*n* = 99) and 60.6% of the LLR (*n* = 80). Underlying liver cirrhosis was present in 25.6% of the OLR (*n* = 40) and 27.2% of the LLR (*n* = 36) patients. Study and patient characteristics are shown in detail in Table 1.

### 3.3. Operative Time

**Figure 2:**
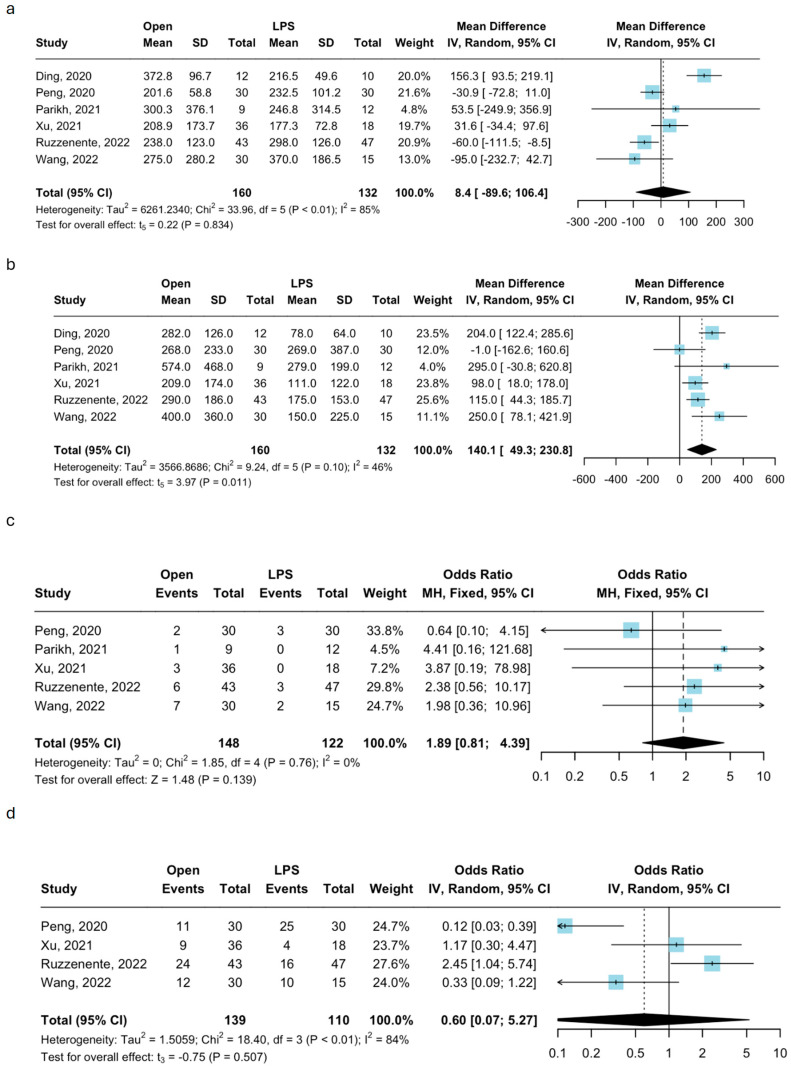

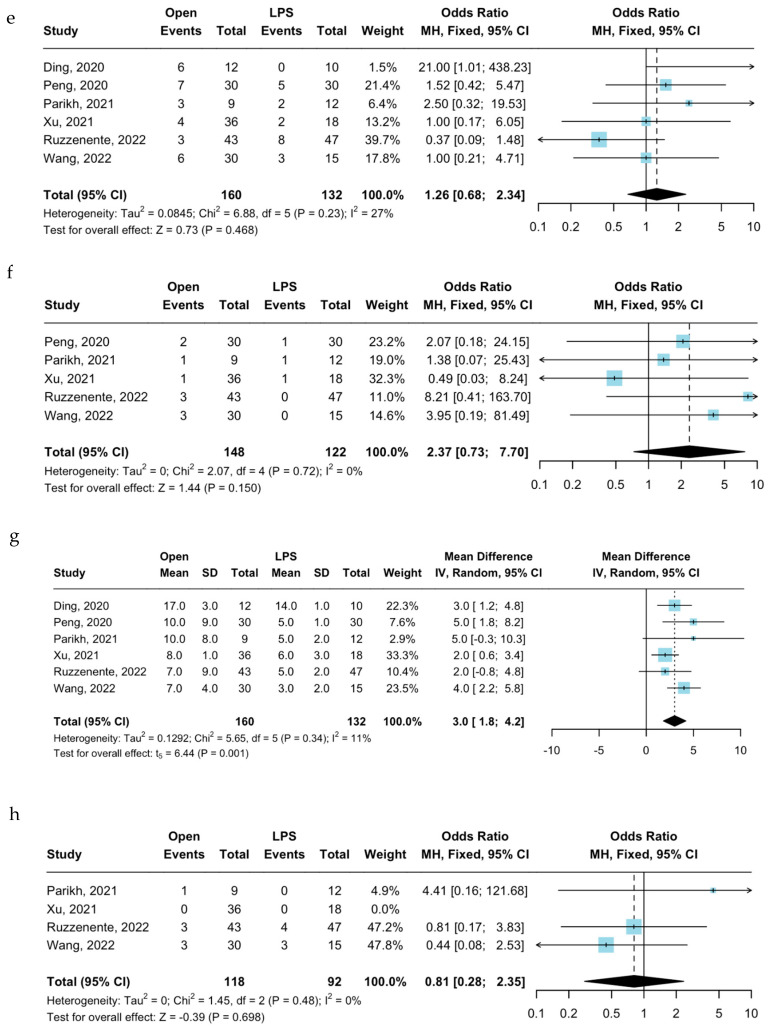
Forest plots depicting the meta-analysis of laparoscopic liver resection versus open liver resection for segment 1. (**a**): operative time; (**b**): estimated blood loss; (**c**): perioperative transfusions; (**d**): Pringle maneuver application; (**e**): total complication rates; (**f**): severe complications rate; (**g**): length of hospital stay; and (**h**): rate of positive resection margins. Ding, 2020 is [23]; Peng, 2020 is [24]; Parikh, 2021 is [25], Xu, 2021 is [26], Ruzzenente, 2022 is [28]; Wang, 2022 is [27]. LPS: Laparoscopy; CI: Confidence Interval; MH: Mantel-Haenszel method; IV: inverse variance.

Six studies with 292 patients (OLR: *n* = 160; LLR: *n* = 132) included in the meta-analysis reported data about operative time [23,24,25,26,27,28]. The pooled MD was comparable between OLR and LLR groups (MD: 8.4, 95% CI 89.6–106.4; *p* = 0.834) (Figure 2a). The heterogeneity was substantial (I^2^ = 85%, *p* < 0.01).

### 3.4. Estimated Blood Loss

All the included six studies with their 292 patients (OLR: *n* = 160; LLR: *n* = 132) showed data regarding intraoperative estimated blood loss (EBL) and were subsequently analyzed [23,24,25,26,27,28]. The pooled MD of the EBL was statistically significantly higher during OLR than LLR (MD: 140.1, 95% CI 49.3–230.8; *p* = 0.011) (Figure 2b). The heterogeneity was moderate (I^2^ = 51%, *p* < 0.10).

### 3.5. Perioperative Transfusions

Data about transfusion rates were reported in five out of six studies, including 270 patients (OLR: *n* = 148; LLR: *n* = 122) [24,25,26,27,28]. The meta-analysis of such studies showed no significant differences in receiving blood transfusions between the two groups (OR: 1.89, 95% CI: 0.81–4.89, *p* = 0.139) (Figure 2c). The heterogeneity was low (I^2^ = 0%, *p* = 0.76).

### 3.6. Pringle Maneuver

The rates of intraoperative Pringle maneuvers were reported by four out of six studies, including 249 patients (OLR: *n* = 139; LLR: *n* = 110) [24,26,27,28]. The meta-analysis of these studies showed a similar risk of receiving intraoperative Pringle maneuvers between the two groups (OR: 0.60, 95% CI: 0.07–5.27; *p* = 0.507) (Figure 2d), with substantial heterogeneity (I^2^ = 84%, *p* < 0.01).

### 3.7. Postoperative Morbidity

Data about the overall rate of postoperative complications were reported for all the 292 patients within the six included studies (OLR: *n* = 160; LLR: *n* = 132), and they were all included in the meta-analysis [23,24,25,26,27,28]. The pooled rates of total postoperative morbidity were 18.1% after OLR (*n* = 29) and 15.1% after LLR (*n* = 20). There were no significant differences in the meta-analysis (OR: 1.26, 95% CI: 0.68–2.34; *p* = 0.468) (Figure 2e); the heterogeneity was low (I^2^ = 27%, *p* = 0.23).

### 3.8. Severe Postoperative Complications

Data about the rates of severe postoperative complications were found in five out of six studies, including 270 patients (OLR: *n* = 148; LLR: *n* = 122) [24,25,26,27,28]. The pooled rates of severe postoperative morbidity after OLR and LLR were 6.7% (*n* = 10) and 2.4% (*n* = 3), respectively. The two approaches showed no significant differences in the meta-analysis (OR: 2.37, 95% CI: 0.73–7.70; *p* = 0.150) (Figure 2f) and low heterogeneity (I^2^ = 0%, *p* = 0.72).

### 3.9. Length of Hospital Stay

Data regarding the postoperative length of stay were reported in all six studies, with 292 patients being included in the meta-analysis (OLR: *n* = 160; LLR: *n* = 132) [23,24,25,26,27,28]. The open approach was associated with a longer postoperative length of stay (MD: 30, 95% CI 1.8–4.2; *p* = 0.001) (Figure 2g). The heterogeneity was low (I^2^ = 11%, *p* = 0.34).

### 3.10. R1 Rate

The rate of microscopically positive resection margins (R1) was shown in four out of six studies, including 210 patients (OLR: *n* = 118; LLR: *n* = 92) [25,26,27,28]. The pooled rate of R1 resections was 5.9% (*n* = 7) after OLR versus 7.6% after LLR (*n* = 7). The analysis showed low heterogeneity (I^2^ = 0%, *p* = 0.48) between with the two techniques (OR: 0.81, 95% CI: 0.28–2.35, *p* = 0.698) (Figure 2h) 

### 3.11. Quality Assessment and Publication Bias

The quality assessment of the included studies can be found in Supplementary Material Figure S1. Moderate-to-high overall risk of bias were found given the nature of the selected studies.

**Figure 3:**
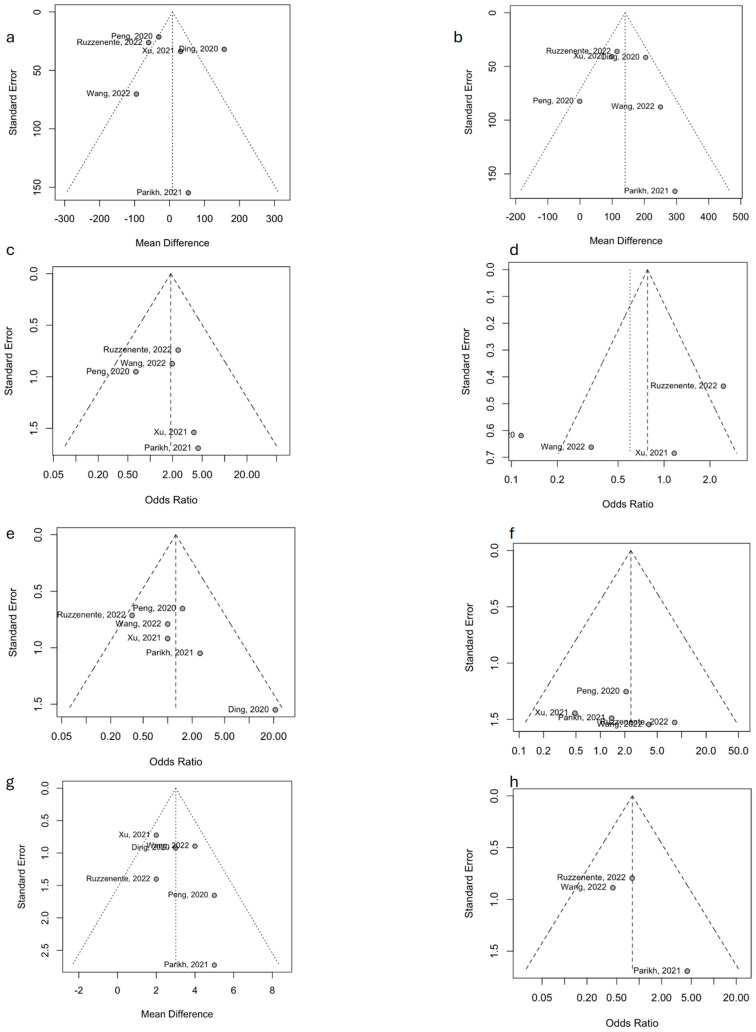
Funnel plots depicting the publication bias. (**a**): operative time; (**b**): estimated blood loss; (**c**): perioperative transfusion; (**d**): Pringle maneuver application; (**e**): total complication rate; (**f**): severe complications rate; (**g**): length of hospital stay; and (**h**): rate of positive resection margins Ding, 2020 is [23]; Peng, 2020 is [24]; Parikh, 2021 is [25], Xu, 2021 is [26], Ruzzenente, 2022 is [28]; Wang, 2022 is [27]. LPS: Laparoscopy; CI: Confidence Interval; MH: Mantel-Haenszel method; IV: inverse variance.

The funnel plots available in Figure 3 show symmetry for all except one tested variable, with only the Pringle maneuver duration showing a risk of publication bias.

### 3.12. Sensitivity Analysis

Outliers and influential cases were checked in case of the I^2^ being greater than 50%. For Pringle maneuver application and operative time, no specific studies were found to be sources of heterogeneity.

## 4. Discussion

This study, for the first time, systematically reviewed the currently available comparative studies on OLR and LLR for R1 resections, and a meta-analysis of the short-term results was performed.

After the first consensus meeting held in Louisville in 2008 and the Morioka consensus in 2014, major LLR and technically difficult resections were still considered innovative procedures in the exploration phase [29,30]. Lesions with difficult locations, such as posterosuperior segments (including S1), were still considered a limitation in some centers for applying MILS. However, both the growing experience and the advances in technologies such as indocyanine green guided fluorescence, 3-dimensional laparoscopy, and even the robotic platforms have pushed the limits of MILS, with a growing number of trials and PSM studies regarding both minor and major hepatectomies, showing some advantages of MILS even in challenging situations [12,31,32,33]. The results of the first randomized trial comparing LLR and OLR for resections in posterosuperior segments have just been published, showing a significantly reduced time to functional recovery in the length of hospital stay after LLR [34]. However, S1 resections were not included. Furthermore, the first comparative study including only isolated caudate lobe resection was published as late as in 2020 to testify the consideration of such resection as even more technically challenging.

This meta-analysis showed lower EBL after LLR of S1 when compared to OLR. This result is coherent with previous comparative studies [23,24,25,26,27,28]. These benefits may be related to the magnified vision of the modern high-resolution cameras in the narrow space including S1, allowing for a precise dissection of blood vessels, bile ducts, and liver parenchyma that is very helpful for the small CL collaterals coming from IVC [28]. Similarly, the caudal view of segment 1 may ease the visualization of such structures when compared to open procedures [35,36]. Moreover, the balance between intra-abdominal pressure and central venous pressure has been proposed as being able to reduce venous bleeding [28].

It is interesting to note that EBL has been reported to be a cause of technical difficulty in MILS, as it is associated with both postoperative morbidity and open conversions [14,37]. In previous studies, intraoperative bleeding was reported to be the most important risk for conversion to open surgery: thus, the reduction in EBL may also be associated with the low conversion rates reported in all the included studies [38]. In particular, when focusing on conversions to open surgery, three out six of the studies included here reported no conversions, with Peng et al. reporting only one conversion (3.3%) and Ruzzenente et al. reporting 6.3% of conversions (*n* = 3), resulting in a pooled rate of 3.4% [24,28]. Furthermore, resection of posterosuperior segments, which also include segment 1, has been advocated to be an important risk factor for conversion from LLR to OLR [38,39]. In this light, the results of this meta-analysis may suggest that the MILS approach for S1 may have a low rate of open conversions in expert centers.

The postoperative length of hospital stay was shorter after LLR, confirming also for caudate lobes the same results as other liver segments [40,41,42]. Indeed, many previous original studies and meta-analyses have systematically shown how minimally invasive surgery can reduce the length of stay after liver resection in difficult positions and in the case of challenging or multiple resections [40,43]. As proposed in previous papers, this result may be due to the reduction in postoperative pain, with subsequent early ambulation, as well as to the early recovery of gastrointestinal movements [7]. Moreover, a reduced length of hospital stay may theoretically lead to an important reduction in the economic costs related to the liver surgery. However, economic evaluations are beyond the aims of our study: further analysis should investigate such an important aspect.

From our results, no statistically significant differences in both overall postoperative morbidity and severe postoperative complications were found after OLR and LLR, with low heterogeneity among the selected works, showing how laparoscopic approach should be considered safe and feasible for S1 resection.

Similarly, the results on R0 resection rates arising from the four included studies showed no differences between the two groups. Similar results showing non-inferiority of LLR relative to OLR in terms of R0 resections have been previously reported for comparisons including all the liver segments [44]. These important results are probably related to the repetitive use of intraoperative ultrasounds, as well as of indocyanine green fluorescence, both helping the surgeon to clearly identify the margins of the liver lesions [45,46].

Finally, the caudate lobe can be classified into three different portions: the caudate process located on the right side of the IVC, the Spiegel’s lobe on the left side of the IVC, and the paracaval portion just in front of the IVC [47]. Although the Spigelian lobe is relatively easy to resect, surgery of the paracaval portion or of the caudate process may be very challenging due to their relationship with the hepato-caval confluence, the biliary duct and the main portal vein bifurcation [48]. According to our results, segment 1 resection may improve some short-term outcomes, lower EBL, and shorten the postoperative length of hospital stay, but surgeons should always pay attention to the potential intraoperative life-threatening massive bleeding caused by injuries to the IVC [49]. Since there are few studies in the literature comparing open and minimally invasive resections, all the subtypes of S1 resections were included in our study, with no regard to the portion of caudate lobe resected. Further study should focus on this aspect. Similarly, different approaches to caudate lobe resections have been described: from the left side, from the right side, and trans-parenchymal [50]. However, the comparison between such different approaches was not within the aims of this meta-analysis. Further studies should be encouraged to investigate the outcomes of each specific access method.

Another aspect that may certainly favor the minimally invasive approach is the unique possibility to integrate the digital visualization system with artificial intelligence (AI) technologies. In this setting, in the future, further studies should evaluate the possibility of applying artificial intelligence to improve surgical outcomes for S1 liver resection, as well as for surgical training. Indeed, the better visualization offered the laparoscopic approach in such a deep field, together with the possibility to record preoperative and intraoperative videos, may be used to teach machines via deep learning methods to further enhance intraoperative experience [51,52,53].

This study has some other limitations. All the included studies were retrospective with a relatively small sample size, potentially leading to selection bias. Similarly, the multicenter nature of the studies with different surgeons‘ skills, the non-standardized surgical technique with the use of different devices, the different underlying liver conditions, the mixed tumor types included, the differences in the histology of the tumor, and the different health care systems may also be considered confounding factors, possibly impacting some outcomes. Nevertheless, propensity score matching was used in four out of six studies to match baseline characteristics between patients undergoing LLR and OLR for S1 resection.

Finally, this is the first systematic review and meta-analysis including only comparative studies regarding the outcomes of LLR versus OLR for caudate lobe. Similarly, it would be interesting in the future to assess the safety and feasibility of a robotic approach for the caudate lobe, given the advantages shown for other posterosuperior segments [54].

## 5. Conclusions

In conclusion, the present systematic review with a meta-analysis demonstrated that LLR of S1 is safe and effective in HPB referral centers and may be superior to OLR in terms of EBL and postoperative length of stay. A tailored approach is paramount to select the best surgical approach based on risk factors for postoperative complications or intraoperative conversions. Future larger prospective studies should confirm such results.

## Figures and Tables

**Table 1:** Patient and tumor characteristics in the included studies. PSM: propensity score matching; BMI: body mass index; SD: standard deviation; OLR: open liver resection; LLR: laparoscopic liver resection; ^#^: median and interquartile range; and *: mean.

Author	Year	Study Type	Approach	Patients, *n*	Age, Mean (SD)	Female Sex, *n* (%)	BMI, Median	Previous Abdominal Surgery, *n* (%)	Underlying Liver Cirrhosis, *n* (%)	Tumor Size, Median in mm	Multifocal Tumor, *n* (%)	Indication Malignant/Benign, *n*
Ding et al. [23]	2020	Retrospective	OLR	12	59.2 (7.8)	8 (75)	NA	1 (8.3)	2 (16.6)	25	N.A.	8/4
			LLR	10	48 (17.1)	5 (50)	NA	0 (0)	2 (20)	60	N.A.	5/5
Peng et al. [24]	2020	Retrospective with PSM	OLR	30	49.7 (14.1)	15 (50)	21.6	8 (26.6)	6 (20)	43	2	14/16
			LLR	30	49 (14.1)	15 (50)	21.8	10 (33.3)	6 (20)	40	2	14/16
Parikh et al. [25]	2021	Retrospective	OLR	9	60 (5.7)	4 (44.4)	24.4	N.A.	3 (33.3)	27	N.A.	9/0
			LLR	12	62.5 (14.7)	3 (25)	24.5	N.A.	6 (50)	20	N.A.	12/0
Xu et al. [26]	2021	Retrospective with PSM	OLR	36	46.6 (11.4)	20 (55.5)	23.3	N.A.	11 (30.5)	46.6	N.A.	12/24
			LLR	18	48.2 (12.5)	10 (55.5)	24.3	N.A.	7 (38.8)	38.3	N.A.	8/10
Ruzzenente et al. [28]	2022	Retrospective with PSM	OLR	43	54.9 (15.7)	19 (44.1)	24.9	16 (37.2)	11 (25.6)	37.7 *	N.A.	30/13
			LLR	42	60.8 (15.9)	22 (52.3)	25.3	19 (45.2)	10 (23.8)	35.7 *	N.A.	30/12
Wang et al. [27]	2022	Retrospective with PSM	OLR	30	63 (14.5) ^#^	10 (33.3)	N.A.	19 (63.3)	9 (30)	26.5	N.A.	26/4
			LLR	15	63 (11) ^#^	6 (40)	N.A.	9 (60)	5 (33.3)	25	N.A.	11/4

## Supplementary Materials

The following supporting information can be downloaded at: https://www.mdpi.com/article/10.3390/jcm14134421/s1.
