# Supplementary figures and images for: Laparoscopic Versus Open Caudate Lobe Resection: A Systematic Review with a Meta-Analysis of Comparative Studies
Source: J Clin Med. 2025 Jun 21;14(13):4421. doi: 10.3390/jcm14134421 (PMC12249736; doi:10.3390/jcm14134421)

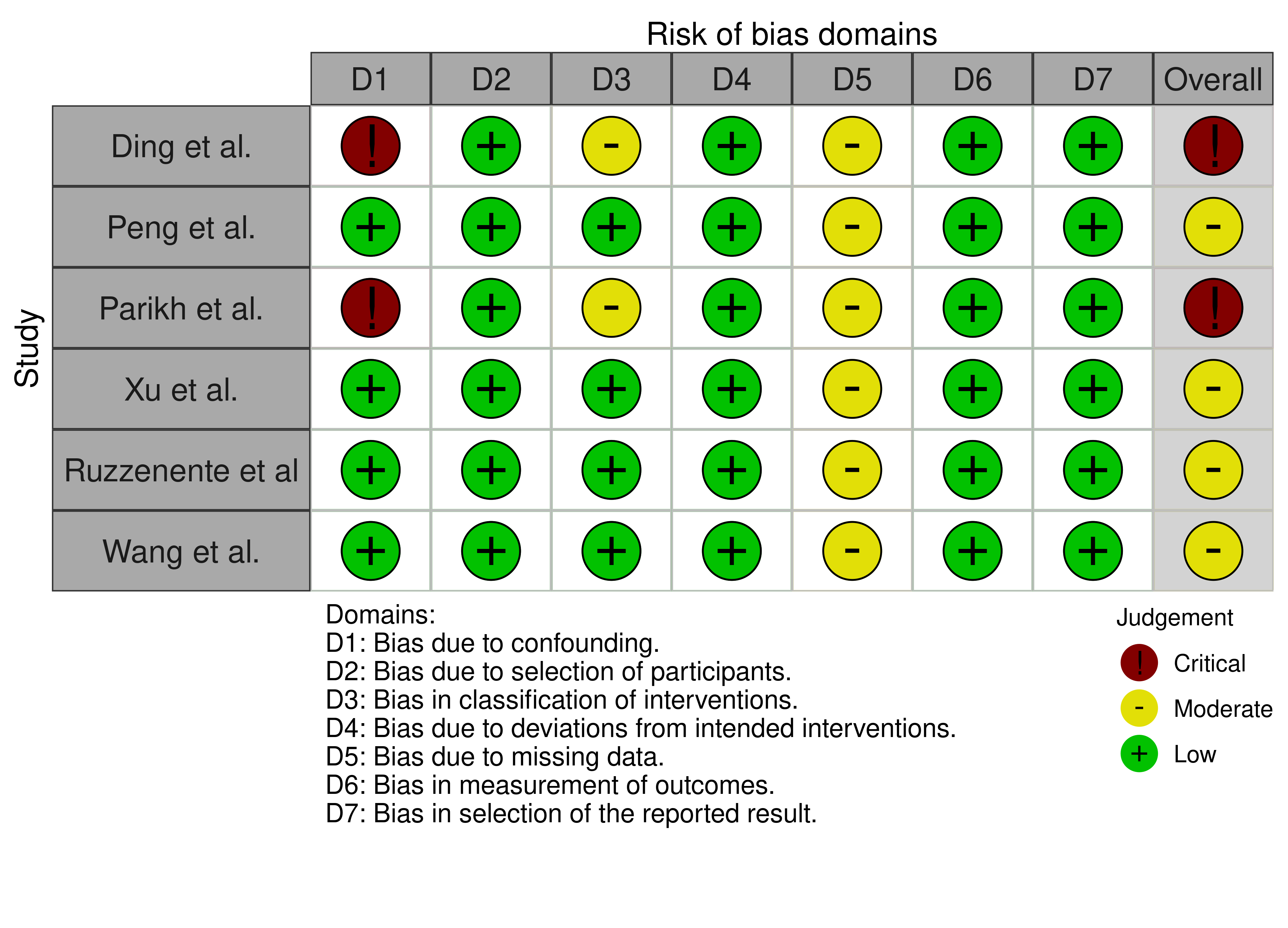

Supplement: Supplementary file 1 [file jcm-14-04421-s001.zip › jcm-3572989-supplementary.tiff]
